# Saireito, a Japanese herbal medicine, alleviates leaky gut associated with antibiotic-induced dysbiosis in mice

**DOI:** 10.1371/journal.pone.0269698

**Published:** 2022-06-15

**Authors:** Sotaro Ozaka, Akira Sonoda, Shimpei Ariki, Mizuki Minata, Naganori Kamiyama, Shinya Hidano, Nozomi Sachi, Kanako Ito, Yoko Kudo, Astri Dewayani, Thanyakorn Chalalai, Takashi Ozaki, Yasuhiro Soga, Chiaki Fukuda, Kazuhiro Mizukami, Shiori Ishizawa, Mitsue Nishiyama, Naoki Fujitsuka, Sachiko Mogami, Kunitsugu Kubota, Kazunari Murakami, Takashi Kobayashi

**Affiliations:** 1 Department of Infectious Disease Control, Faculty of Medicine, Oita University, Yufu, Oita, Japan; 2 Department of Gastroenterology, Faculty of Medicine, Oita University, Yufu, Oita, Japan; 3 Tsumura Advanced Technology Research Laboratories, Tsumura & Co., Ibaraki, Japan; 4 Tsumura Kampo Research Laboratories, Tsumura & Co., Ibaraki, Japan; Toho University Graduate School of Medicine, JAPAN

## Abstract

Antibiotics disrupt normal gut microbiota and cause dysbiosis, leading to a reduction in intestinal epithelial barrier function. Disruption of the intestinal epithelial barrier, which is known as “leaky gut”, results in increased intestinal permeability and contributes to the development or exacerbation of gastrointestinal diseases such as inflammatory bowel disease and irritable bowel syndrome. We have previously reported on a murine model of intestinal epithelial barrier dysfunction associated with dysbiosis induced by the administration of ampicillin and vancomycin. Saireito, a traditional Japanese herbal medicine, is often used to treat autoimmune disorders including ulcerative colitis; the possible mechanism of action and its efficacy, however, remains unclear. In this study, we examined the efficacy of Saireito in our animal model for leaky gut associated with dysbiosis. C57BL/6 mice were fed a Saireito diet for the entirety of the protocol (day1-28). To induce colitis, ampicillin and vancomycin were administered in drinking water for the last seven consecutive days (day22-28). As previously demonstrated, treatment with antibiotics caused fecal occult bleeding, cecum enlargement with black discoloration, colon inflammation with epithelial cell apoptosis, and upregulation of pro-inflammatory cytokines. Oral administration of Saireito significantly improved antibiotics-induced fecal occult bleeding and cecum enlargement by suppressing inflammation in the colon. Furthermore, Saireito treatment ensured the integrity of the intestinal epithelial barrier by suppressing apoptosis and inducing cell adhesion proteins including ZO-1, occludin, and E-cadherin in intestinal epithelial cells, which in turn decreased intestinal epithelial permeability. Moreover, the reduced microbial diversity seen in the gut of mice treated with antibiotics was remarkably improved with the administration of Saireito. In addition, Saireito altered the composition of gut microbiota in these mice. These results suggest that Saireito alleviates leaky gut caused by antibiotic-induced dysbiosis. Our findings provide a potentially new therapeutic strategy for antibiotic-related gastrointestinal disorders.

## Introduction

Although antibiotics are the most frequently used clinical drugs for treating bacterial infections, they can also disrupt normal gut microbiota and cause dysbiosis [1]. It is well known that dysbiosis increases the risk of gastrointestinal disorders including antibiotic-associated colitis and inflammatory bowel disease (IBD) [2, 3]. Recently, it has been reported that dysbiosis induced by antibiotics reduces intestinal epithelial barrier function [4–6]. The physical integrity of epithelial barrier is maintained by only a single layer of epithelial cells that are held together by cell adhesion proteins. Disruption of the physical barrier results in increased intestinal permeability, which in turn facilitates the translocation of endotoxin and other bacterial toxins from the intestinal lumen to the bloodstream, leading to the activation of immune responses [7]. This condition is known as “leaky gut”. Gastrointestinal diseases such as IBD and irritable bowel syndrome (IBS) as well as non-gastrointestinal diseases such as type 1 diabetes, multiple sclerosis, systemic lupus erythematosus (SLE), and non-alcoholic fatty liver disease (NAFLD) may arise or be exacerbated by leaky gut [8–11].

We have previously reported a murine model of physical epithelial barrier dysfunction associated with dysbiosis induced by administration of antibiotics. Oral administration of ampicillin (ABPC) and vancomycin (VCM) induced fecal occult bleeding (FOB), an enlarged cecum, production of pro-inflammatory cytokines, a reduction in Ki-67-positive cells and an increase in apoptotic cells accompanied by reduced diversity of gut microbiota and lowered levels of short chain fatty acids (SCFAs) and glutamic acid in the colon [12].

Given the impact of leaky gut on multiple diseases, protecting the integrity of the intestinal epithelial barrier is extremely important for the maintenance of gut homeostasis. Saireito (TJ-114, SRT), a traditional Japanese herbal (Kampo) medicine, is composed of 12 medicinal herbs and is often used to treat autoimmune disorders such as rheumatoid arthritis, SLE and ulcerative colitis [13–15], however, the potential mechanism of action and its efficacy remains unclear. Recently, animal experiments have demonstrated the anti-inflammatory effects of Saireito in chemotherapy-induced mucositis and IBD [16, 17]. However, the effects of Saireito on antibiotic-associated colitis, dysbiosis, and intestinal epithelial barrier dysfunction have not been described. Thus, we sought to investigate the effect of Saireito on leaky gut associated with dysbiosis in mice treated with ABPC and VCM (A+V-treated mice).

Here we show that oral administration of Saireito significantly improves antibiotic-induced FOB and cecum enlargement by suppressing inflammation in the colon. Saireito treatment ensures the physical integrity of the intestinal epithelial barrier by suppressing apoptosis and inducing the production of cell adhesion proteins in intestinal epithelial cells, which in turn decreases intestinal epithelial permeability. Hence, Saireito alleviates leaky gut caused by antibiotic-induced dysbiosis.

## Materials and methods

### Drugs and chemicals

Ampicillin (ABPC) and vancomycin (VCM) were purchased from Wako (Osaka, Japan). Saireito (TJ-114, SRT) was obtained as a powder from Tsumura & Co. (Ibaraki, Japan). Saireito or Daikenchuto (TJ-100) were included in mouse diet AIN76A, a defined diet (Oriental Yeast Co., ltd. Tokyo, Japan), at 15 g Saireito or Daikenchuto/kg of diet (1.5% wt/wt) (Oriental Yeast). The dosage of Saireito was based on previous animal experiments [16]. Hemoccult for fecal occult blood test was purchased from Beckman Coulter (Brea, CA, USA).

### Cell culture

A mouse colon carcinoma cell line, CMT93, was purchased from ECACC (Salisbury, UK). CMT93 cells were cultured in Dulbecco’s modified Eagle medium (DMEM) containing 10% FCS, 100 U/mL Penicillin, 100 μg/mL Streptomycin (Nacalai tesque, Kyoto, Japan) and 2 mM L-Alanyl-L-glutamine (Nacalai tesque). Saireito (Tsumura & Co) was suspended in distilled water, sterilized by boiling at 95°C, passed through a 0.45 μm filter, and then added to the cultures at final concentrations of 1 or 3 μg /mL. CMT93 cells were cultured with 1 or 3 μg /mL of Saireito for 24 hours. Then, cells were harvested and total RNA was extracted using TRI Reagent^®^ (Sigma-Aldrich, St. Louis, MO, USA) for carrying out quantitative RT-PCR analysis.

### Animals and ethics statement

Seven-week-old female C57BL/6 mice were purchased from Japan SLC (Hamamatsu, Japan). Mice were maintained in a specific pathogen-free facility under conditions of constant temperature (24 ± 1°C), humidity (50–60%), and a 12-hour light-dark cycle with free access to food and water. All experiments using these mice were approved by and performed according to the guidelines of the Oita University Animal Ethics Committee (approval number: 180901A). A protocol was designed for 28 days and mice were assigned to three groups. Mice were fed a normal diet or a 1.5% Saireito diet during this protocol (day1-28). To induce colitis, 1 g/L ABPC and 0.5 g/L VCM (A + V) were administered in drinking water for the last seven consecutive days (day22-28). In the first group, designated as the normal control, mice were given a normal diet and normal drinking water (Normal group). The second group of mice, designated as the ABT (antibiotic therapy) control, were given a normal diet and A + V solution in their drinking water (ABT group). The third group, designated as the ABT + SRT group, were given a 1.5% Saireito diet and A + V solution in their drinking water (ABT + SRT group). Fecal occult blood test was carried out on day21 (before administration of A + V solution), day24 (three days after administration of A + V solution), and day28.

### Histological evaluation and TUNEL staining analysis

Mice were sacrificed by cervical dislocation and the large intestine was removed on day28. 4 μm sections of 10% formaldehyde-fixed/paraffin embedded colons were stained with hematoxylin and eosin (H&E). The severity of colon inflammation was evaluated using both proximal and distal colon sections with a modified histological scoring system [18] as follows: epithelial cell damage (0: no damage; 1: focal loss of goblet cells; 2: diffuse loss of goblet cells; 3: focal loss of crypts; 4: diffuse loss of crypts), cell infiltration (0: no increase; 1: around base of the crypts; 2: along layer of muscularis mucosae; 3: mucosal layer; 4: mucosal and submucosal layer), ulcer (0: no ulcer; 1: focal erosion; 2: diffuse shallow ulcer of epithelial surface or focal ulcer of mucosal layer; 3: diffuse ulcer involving whole mucosal layer). Intestinal epithelial cell apoptosis was detected by TUNEL staining using TACS2^®^ TdT-DAB In Situ Apoptosis Detection Kit (Trevigen, Gaithersburg, MD, USA), according to the manufacturer’s instructions. Apoptotic cells were calculated by counting total number of TUNEL positive cells in at least four sections at magnification of 400×. Immunohistochemistry was performed using 2 *μ*m sections of paraffin-embedded colon tissue. We used primary antibodies against ZO-1 (Gene Tex, Irvine, CA, USA), occludin (Abcam, Cambridge, UK), and E-cadherin (Cell Signaling Technology, Danvers, MA, USA). DAKO EnVision^™^+ (Rabbit) (Agilent Technologies) was used as a secondary antibody. Sections were subsequently counterstained with hematoxylin. All histological evaluation was performed in a blind fashion.

### Quantitative real-time reverse transcription polymerase chain reaction (RT-PCR)

Total RNA was extracted using TRI Reagent^®^ (Sigma-Aldrich), purified using a PureLink RNA Mini Kit (Thermo Fisher Scientific Inc., Waltham, MA, USA) and then reverse transcribed using a Verso cDNA Synthesis Kit (Thermo Fisher Scientific Inc.). Quantitative RT-PCR was performed using a real-time PCR machine (LightCycler 96, Roche Diagnostics, Rotkreuz, Switzerland) with a KAPA SYBR FAST qPCR Kit (Kapa Biosystems, Wilmington, MA, USA). The relative mRNA levels were normalized to β-actin, and all data was analyzed by LightCycler software (Roche Diagnostics). The sequences of primers used in this study are shown in S1 Table.

### Gas chromatograph-mass spectrometry analysis (GC-MS)

Metabolites in the cecal contents were analyzed by GC/MS-TQ8040 (Shimazu, Kyoto, Japan) with a BPX-5 column (30 m × 0.25 mm i.d.; film thickness 1.00 *μ*m, Trajan Scientific and Medical, Vic., Australia) for short chain fatty acids (SCFAs), and a DB-5 column (30 m × 0.25 mm i.d.; film thickness 1.00 *μ*m, J&W Scientific Inc, Folsom, CA, USA) for other previous described metabolites [12]. Mass spectrum peaks were detected using GCMSsolution software (Shimazu), and the retention time correction of peaks was performed based on the retention time of a standard alkane series mixture (C9 to C33). Metabolites were detected by Smart Metabolites Database (Shimazu) which registered 12 spectrums for BPX-5 column and 475 spectrums for DB-5 column.

### DNA extraction and 16S rRNA gene metagenome sequencing analysis

The bacterial genomic DNA was isolated using the standard protocol with some modifications [19]. DNA from mouse feces was extracted using a phenol/chloroform/isoamyl alcohol method. The preparation of 16S rRNA gene metagenome library for MiSeq (Illumina, Inc., San Diego, USA) was performed according to the manufacturer’s protocol. Briefly, 10 ng of DNA template was amplified using Advantage-HF 2 PCR kit (Takara Bio Inc., Shiga, Japan) with universal primers for the 16S rRNA v3-v4 region (forward primer: 5’ TCGTCGGCAGCGTCAGATGTGTATAAGAGACAGCCTACGGGNGGCWGCAG 3’, reverse primer: 5’ GTCTCGTGGGCTCGGAGATGTGTATAAGAGACAGGACTACHVGGGTATCTAATCC 3’). Subsequently, index sequences for each sample were added to both ends of the purified PCR fragments. The concentrations of each amplicon were measured by Quant-iT PicoGreen dsDNA Assay Kit (Thermo Fisher Scientific, Inc.) and mixed equally. The library was applied to MiSeq Reagent Kit v3 (Illumina, Inc.) and the sequence was determined using the manufacturer’s standard protocol. Sequence data was processed as follows using the 16S rRNA sequence analysis pipeline, QIIME 1.9.0 [20]. Initially, both sequence reads were joined and sequences with a Phred quality score below 20 removed. Chimera elimination by Usearch was performed to remove contaminated sequences. The open reference OTU picking was performed against Greengenes 13_8 97% OTU representative sequences. A summary of taxonomy in each sample was obtained using the script ‘summarize_taxonomy_through_plots.py’ in QIIME 1.9.0.

### Quantitative polymerase chain reaction (qPCR) for 16S rRNA gene

qPCR for 16S rRNA gene was performed using universal primer: 515F (5′-GTGCCAGCMGCCGCGGTAA-3′) and 806R (5′-GGACTACHVGGGTWTCTAAT-3′) [21]. All primers were purchased from Thermo Fisher Scientific, Inc. as custom primers. DNA extracted from stool samples was used as a template. Reactions were performed according to standard protocol using a SYBR Green PCR Kit (Thermo Fisher Scientific, Inc.) and QuantStudio 7 Flex Real-Time PCR system (Thermo Fisher Scientific, Inc.).

### Measurement of intestinal permeability

Mice were fasted for 4 hours and then administered FITC-dextran (4 kDa MW, 0.6 mg/g body weight) (Sigma-Aldrich) by oral gavage. 4 hours later, blood samples were collected using the heart puncture method. Intestinal permeability was evaluated by measuring the fluorescence intensity of serum FITC-dextran at an excitation/emission wavelength of 485/528 nm using a microplate reader (Infinite 200 PRO, TECAN, Männedorf, Switzerland).

### Statistical analysis

All data are presented as mean ± SEM. Differences between two groups were analyzed by student’s *t* test. Multiple comparisons were analyzed by one-way ANOVA followed by Tukey’s multiple comparisons test or Dunnett’s test. Alpha diversity metrics and beta diversity analysis in cecal contents were calculated using the QIIME 1.9.0. Univariate analysis between two groups was performed with the Mann-Whitney *U* test using GraphPad Prism7 software (GraphPad Software, San Diego, CA, USA). The graphs were visualized by Excel Software and GraphPad Prism 7 software. *P* values less than 0.05 were considered statistically significant in all experiments. In figures, *P < 0.05, **P < 0.01, ***P < 0.001, ****P < 0.0001.

## Results

### Saireito treatment improves antibiotic-induced fecal occult bleeding and cecum enlargement

**Fig 1:**
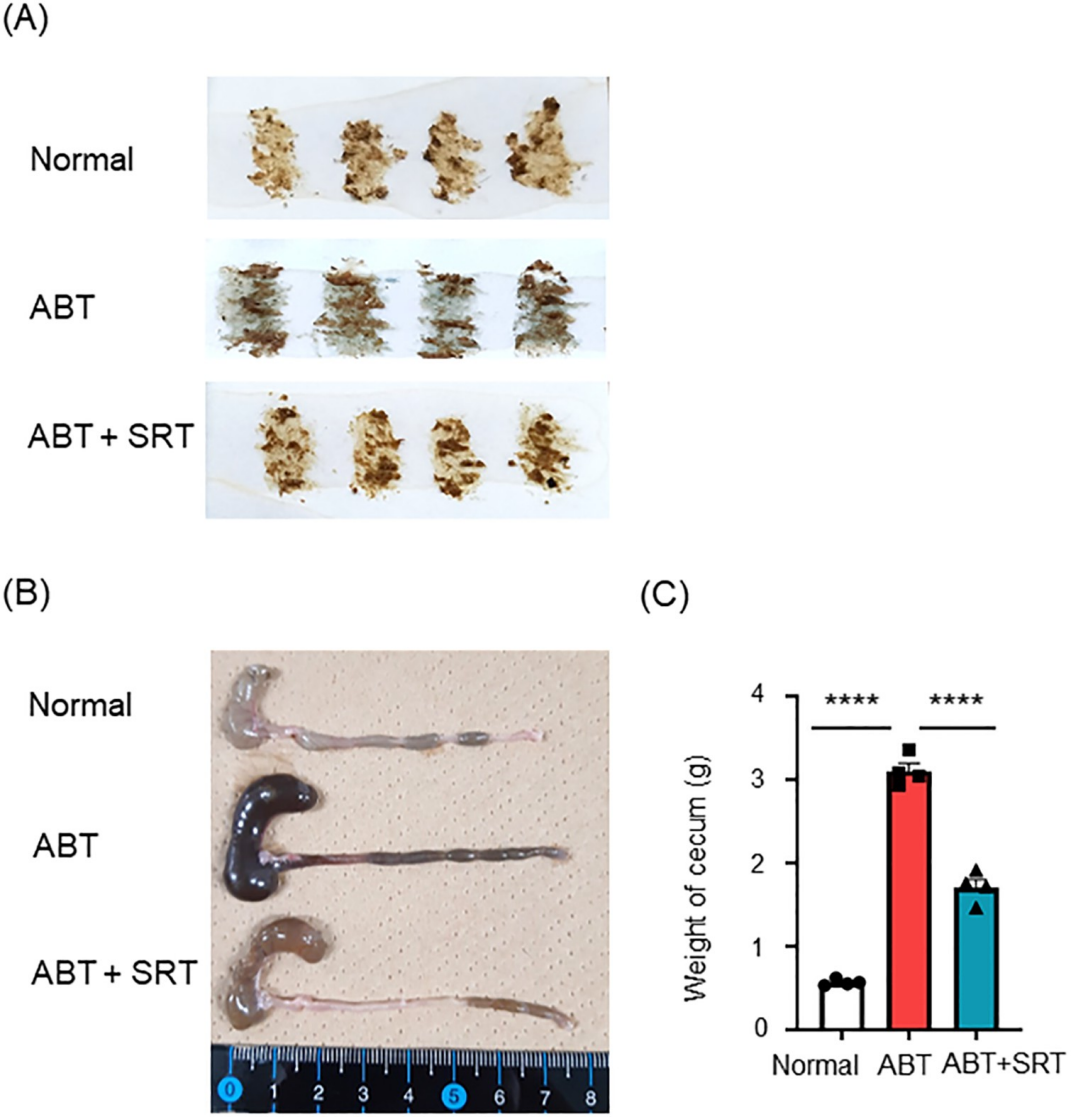
Effect of Saireito on antibiotic-induced fecal occult bleeding and cecum enlargement. Mice in the normal group were given a normal diet and normal drinking water; ABT group mice were given a normal diet and A + V solution in drinking water for the last 7 days; ABT + SRT group mice were fed a 1.5% Saireito diet until the last day and given A + V solution in drinking water for the last 7 days. (A) Images of fecal occult bleeding test (FOBT) on day 28. If FOBT is positive, it will turn blue. (B) Graph shows the images of large intestines. (C) Bar graphs show the weight of cecums. Statistical analysis was carried out using one-way ANOVA followed by Tukey’s multiple comparisons test. Data are shown as mean ± SEM (n = 4, each group). ****P < 0.0001.

Administration of ABPC and VCM (A + V) to mice for seven days induced fecal occult bleeding (FOB) (Table 1 and Fig 1A).

**Table 1:** Number of fecal occult bleeding positive mice.

	day21	day24	day28
Normal	0 / 4	0 / 4	0 / 4
ABT	0 / 4	4 / 4	4 / 4
ABT+Saireito	0 / 4	1 / 4	0 / 4
ABT+Daikenchuto	0 / 4	4 / 4	4 / 4

The cecum from antibiotic-treated mice were enlarged and the contents of the cecum and colon changed to a dark color (Fig 1B). The weight of the cecum from antibiotic-treated mice was about three times heavier than that of normal mice (Fig 1C). In the present study, treatment with a diet incorporated with Saireito or Daikenchuto was started 21 days prior to administration of A + V and continued to the end of experiment. While administration of Daikenchuto failed to inhibit FOB, Saireito prevented FOB in all mice treated with antibiotics for seven days (Table 1 and Fig 1A). In addition, cecum enlargement and the dark color tone were improved, and the weight of the cecum was significantly decreased in the ABT + SRT group (Fig 1B and 1C).

### Saireito treatment reduces antibiotic-induced colon inflammation

**Fig 2:**
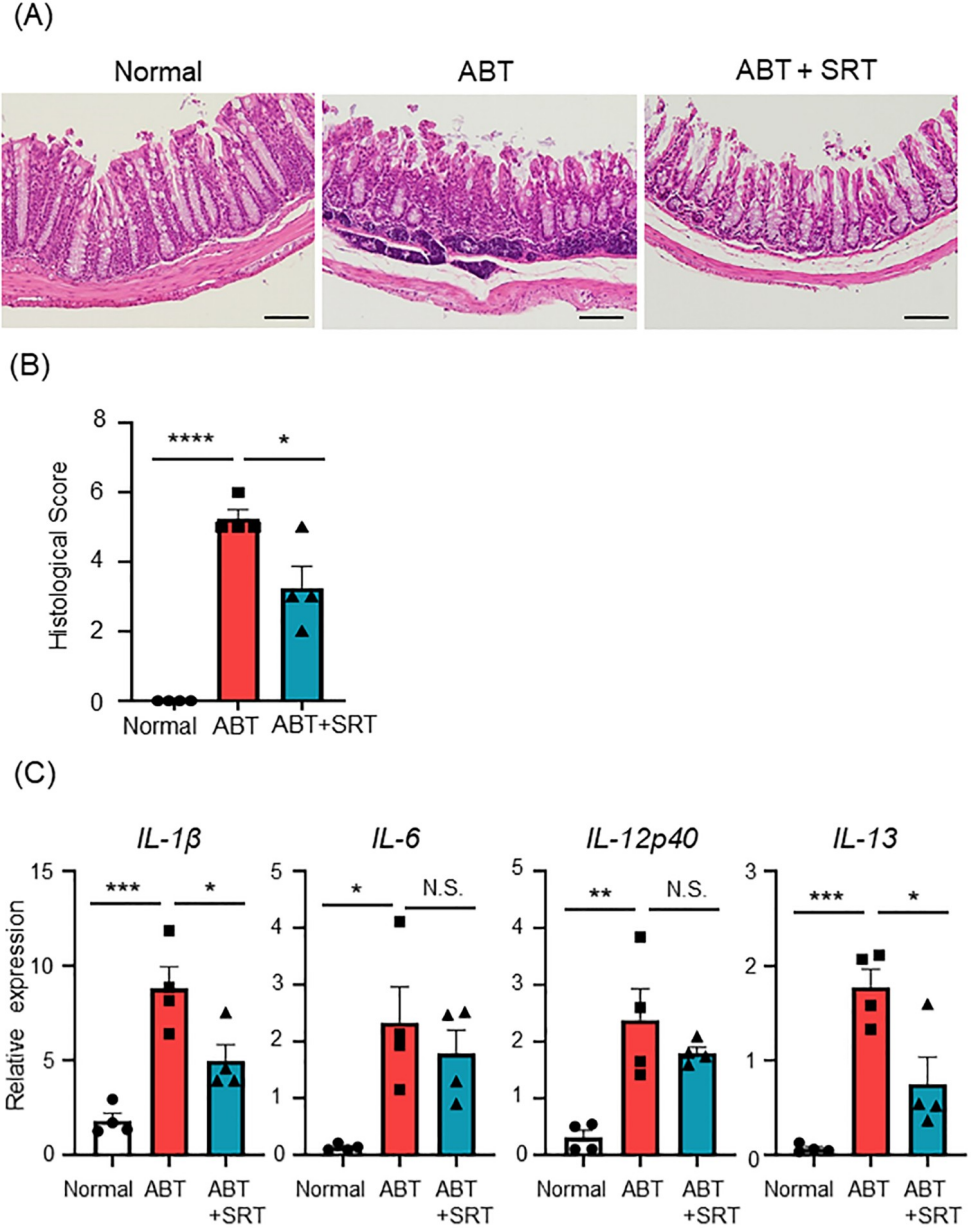
Effect of Saireito on antibiotics-induced colon inflammation. Mice in normal group were given a normal diet and normal drinking water; ABT group mice were given a normal diet and A + V solution in drinking water for the last 7 days; ABT + SRT group mice were fed a 1.5% Saireito diet until the last day and given A + V solution in drinking water for the last 7 days. (A) The colon was excised on day 28, sectioned, and stained with H&E (×200). (B) Graph shows the histological score. (C) mRNA expression level of pro-inflammatory cytokines was determined by quantitative RT-PCR. Statistical analysis was carried out using one-way ANOVA followed by Tukey’s multiple comparisons test. Results are expressed as the mean ± SEM (n = 4, each group). * *P* < 0.05, ** *P* < 0.01, *** *P* < 0.0001, **** *P* < 0.0001, and NS: not significant.

Histological analysis revealed that the administration of A + V caused a loss of goblet cells and infiltration of inflammatory cells into the colon (Fig 2A). Treatment with Saireito was shown to ameliorate the inflammatory changes induced by these antibiotics (Fig 2A and 2B). We next investigated the mRNA expression levels of pro-inflammatory cytokines in the colonic tissue. mRNA levels of IL-1β, IL-6, IL-12p40, and IL-13 were significantly up-regulated in the colon of A + V-treated mice when compared to normal mice. The expression levels of IL-1β and IL-13 in the colons of the ABT group were significantly improved by treatment with Saireito, while mRNA levels of IL-6 and IL-12p40 remained up-regulated (Fig 2C). These results suggest that Saireito has a restorative effect on antibiotic-induced colon inflammation in mice.

### Saireito treatment protects the physical epithelial barrier by suppressing apoptosis of intestinal epithelial cells

**Fig 3:**
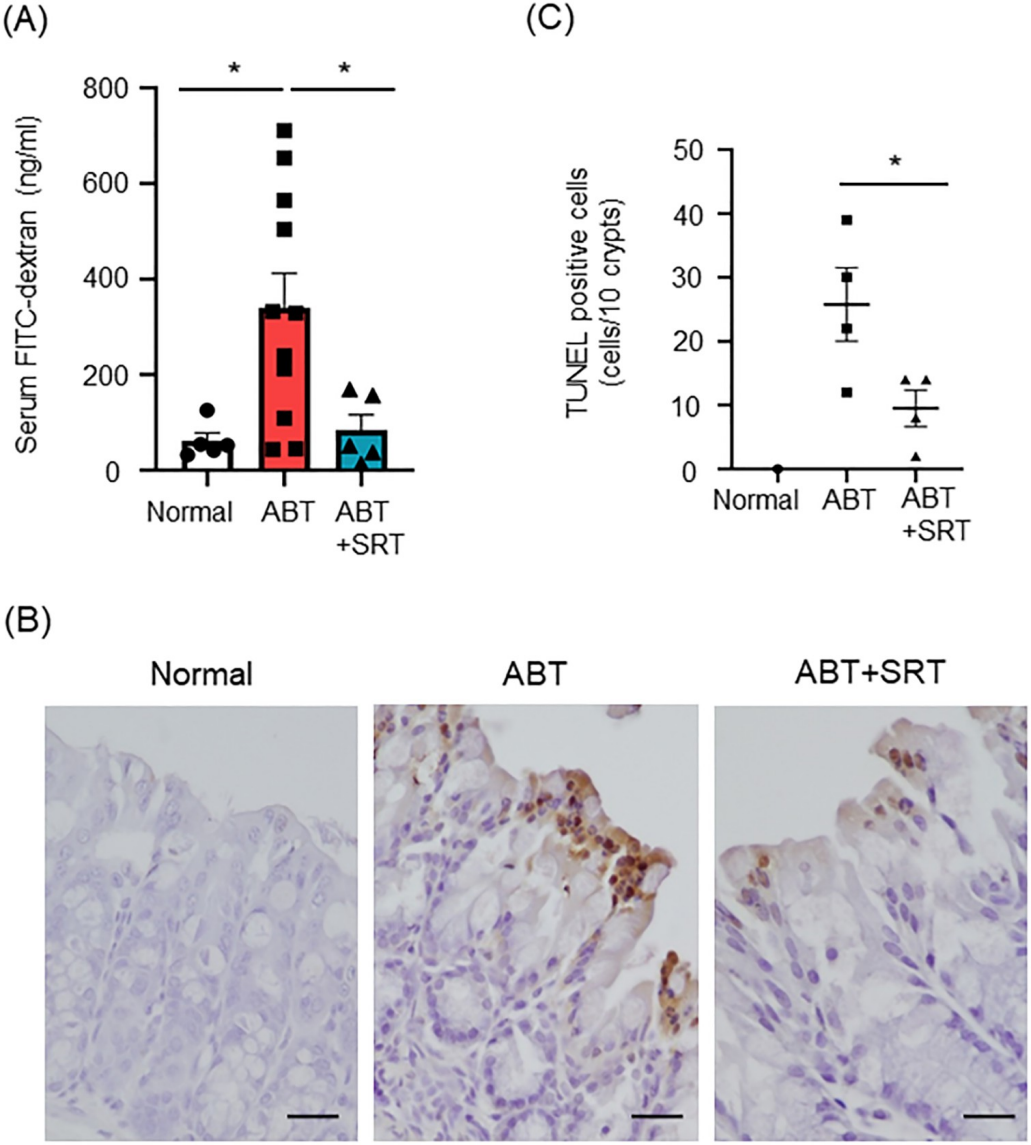
Effect of Saireito on antibiotic-induced colonic epithelial barrier dysfunction and cell apoptosis in the colon. Normal group mice were given a normal diet and normal drinking water; ABT group mice were given a normal diet and A + V solution in drinking water for the last 7 days; ABT + SRT group mice were fed a 1.5% Saireito diet until the last day and given A + V solution in drinking water for the last 7 days. (A) Graph shows FITC-dextran levels in the serum of each mouse. One representative of two independent experiments (n = 5, normal group. n = 11, ABT group and n = 5, ABT + SRT group). (B) Graph shows TUNEL staining of the colon (× 400). (C) Graph shows the number of TUNEL positive apoptotic cells in each section (n = 4, each group). Data were analyzed by one-way ANOVA followed by Tukey’s multiple comparisons test and student *t* test. Data are shown as mean ± SEM. * *P* < 0.05.

The intestinal epithelial barrier is important for intestinal homeostasis and its dysfunction is implicated in the development of intestinal inflammation [22]. We next examined intestinal epithelial permeability to assess the effects of Saireito on the intestinal epithelial barrier in the context of antibiotic induced colitis. Mice were gavaged with FITC-dextran and intestinal permeability was evaluated by serum FITC-dextran levels. On day 28, the serum FITC-dextran levels were significantly increased in the ABT group compared with the normal group. Pre-treatment with Saireito significantly suppressed the serum FITC-dextran levels to the same levels as seen in normal control (Fig 3A). This data indicates that Saireito decreased intestinal epithelial permeability, which was increased in mice treated with antibiotics.

We have previously reported that administration of antibiotics increases apoptosis of intestinal epithelial cells [12]. In the present study, we used TUNEL staining to determine whether Saireito could suppress the apoptosis of intestinal epithelial cells. The number of TUNEL positive cells increased about 25-fold in the ABT group compared to the normal group (Fig 3B and 3C). Following treatment with Saireito, the number of TUNEL positive cells was significantly reduced (Fig 3B and 3C). These results suggest that Saireito protects the physical epithelial barrier by reducing apoptosis of intestinal epithelial cells.

### Saireito enhances the expression of cell adhesion proteins in the colon

**Fig 4:**
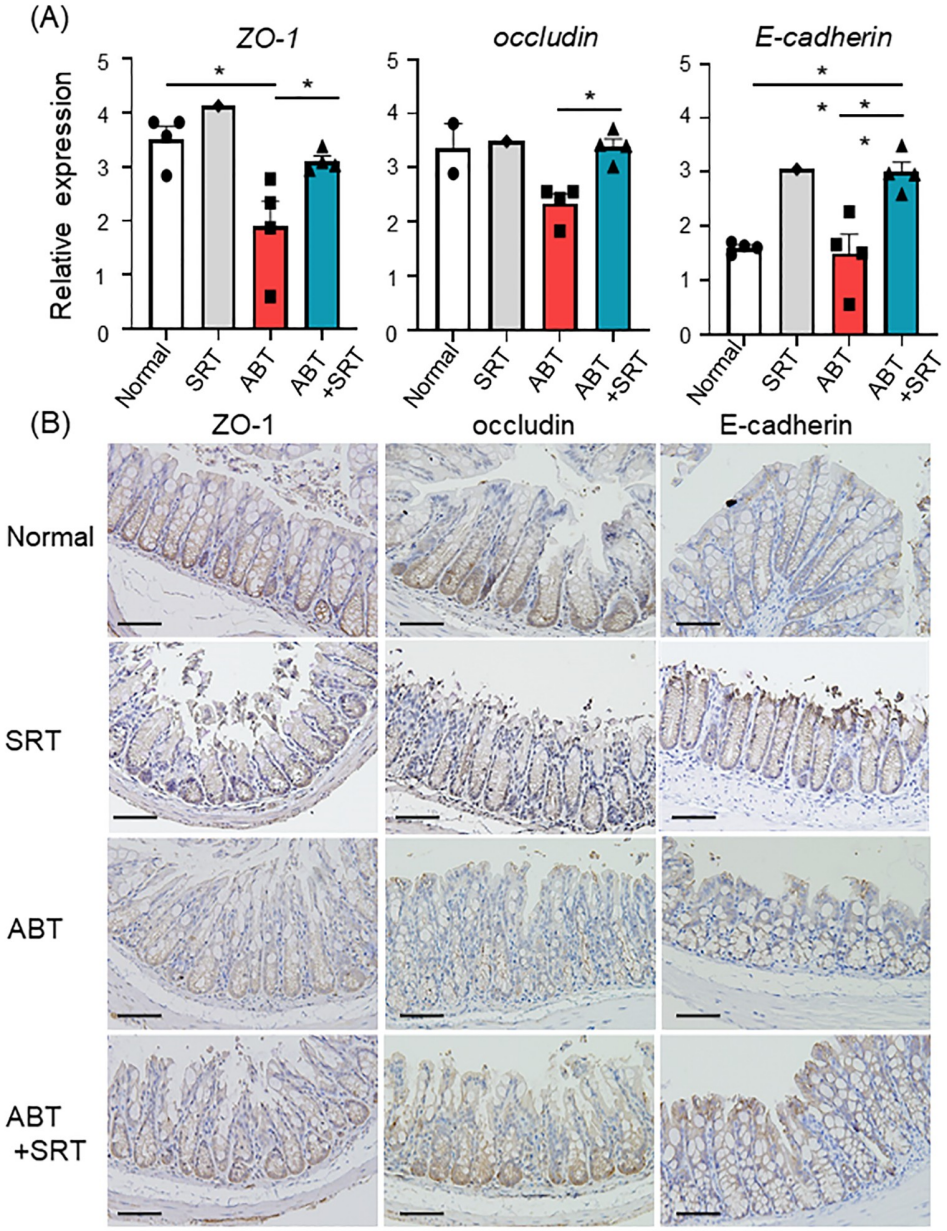
Effect of Saireito on expression of cell adhesion proteins. Normal group mice were given a normal diet and normal drinking water; ABT group mice were given a normal diet and A + V solution in drinking water for the last 7 days; ABT + SRT group mice were fed a 1.5% Saireito diet until the last day and given A + V solution in drinking water for the last 7 days. (A) mRNA analysis of cell adhesion proteins. Statistical analysis was carried out using one-way ANOVA followed by Tukey’s multiple comparisons test. Data are shown as mean ± SEM (n = 4, each group). * *P* < 0.05, **P < 0.01. (B) The figure shows immunohistochemistry for ZO-1, occludin, and E-cadherin (×200).

**Fig 5:**
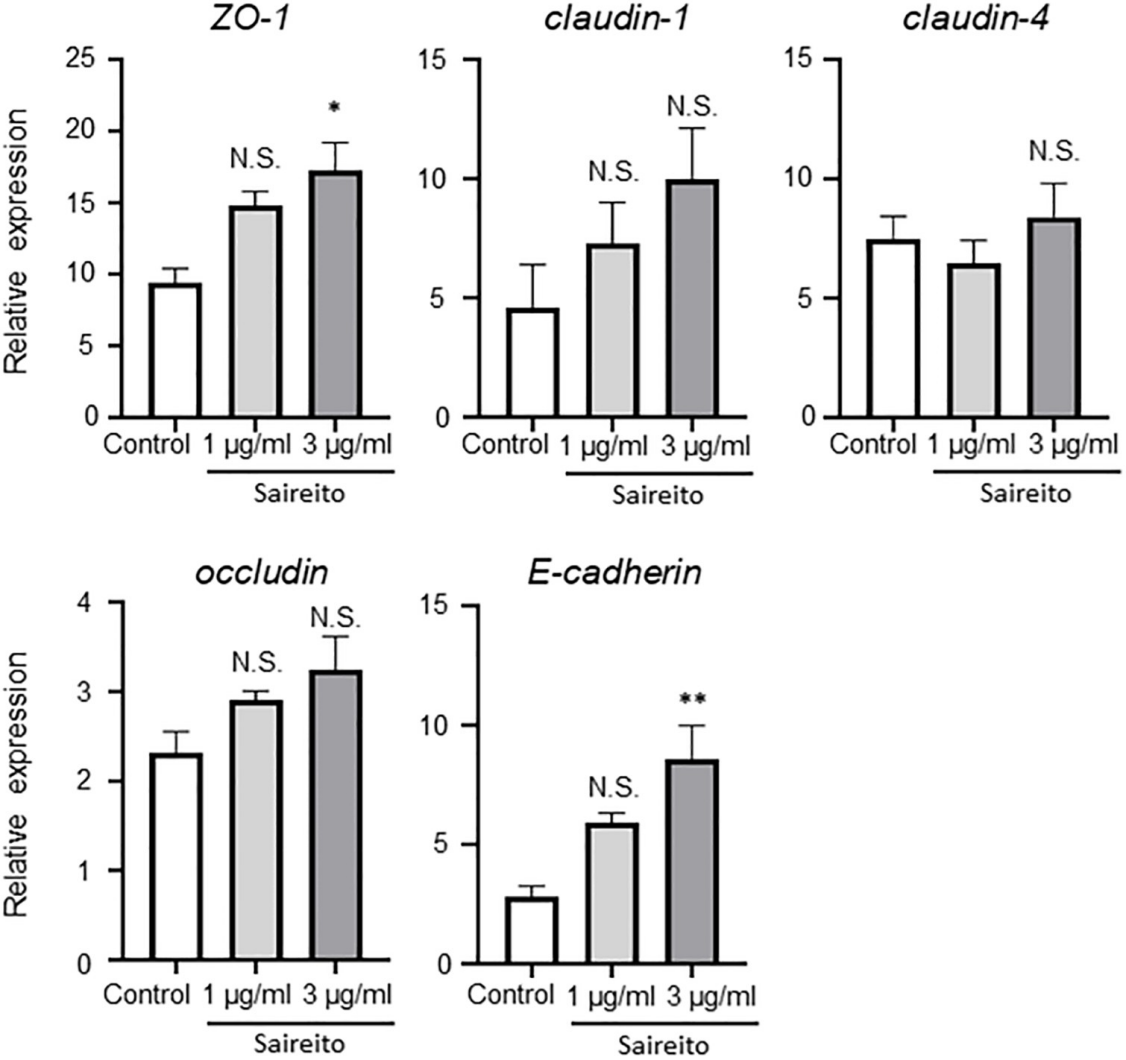
Effect of Saireito co-treatment on the induction of cell adhesion protein in vitro. (A) mRNA analysis of cell adhesion proteins *in vitro*. The concentrations of Saireito were 1 *μ*g/ml or 3 *μ*g/ml. Statistical analysis was carried out using Dunnett’s test. Data are shown as mean ± SEM (n = 3, each group). * *P* < 0.05, **P < 0.01 vs Control.

Cell adhesion proteins are also important components of the physical epithelial barrier [23]. Because of this, we examined mRNA expression levels of cell adhesion proteins in the intestine and found a significant decrease in their expression levels except for claudin-1 in the ABT group compared to the normal group. Interestingly, pretreatment with Saireito increased mRNA expression levels of ZO-1, occludin, and E-cadherin (Fig 4A). mRNA levels of claudin-4 were also increased but not with statistical significance in the ABT + SRT group (S1 Fig). We then performed immunohistochemistry for cell adhesion proteins. Consistent with the results of quantitative real-time PCR, ZO-1 and occludin were weakly expressed in the ABT group, while expression was improved in the ABT + SRT group (Fig 4B). The expression of E-cadherin was enhanced in the ABT + SRT group (Fig 4B). To examine whether Saireito directly induces these adhesion molecules, CMT93 cells, a colon carcinoma cell line, were cultured in the presence of Saireito. As shown in Fig 5, mRNA for ZO-1 and E-cadherin was significantly increased after 24 hours in a dose-dependent manner (Fig 5).

Secretory leukocyte protease inhibitor (SLPI) and lactoferrin are known as antimicrobial peptides and are components of the gut chemical barrier [24]. In addition, MUC2, a component of the colonic mucous layer, plays an important role in the maintenance of gut barrier function [25]. The mRNA expression level of SLPI was slightly increased but not to a statistically significant level, and lactoferrin and MUC2 were reduced with A + V treatment or Saireito pretreatment (S1 Fig).

These results indicate that Saireito protects the physical epithelial barrier by maintaining cell adhesion proteins.

### The effect of Saireito on gut microbiota and fecal metabolites in antibiotic-treated mice

**Fig 6:**
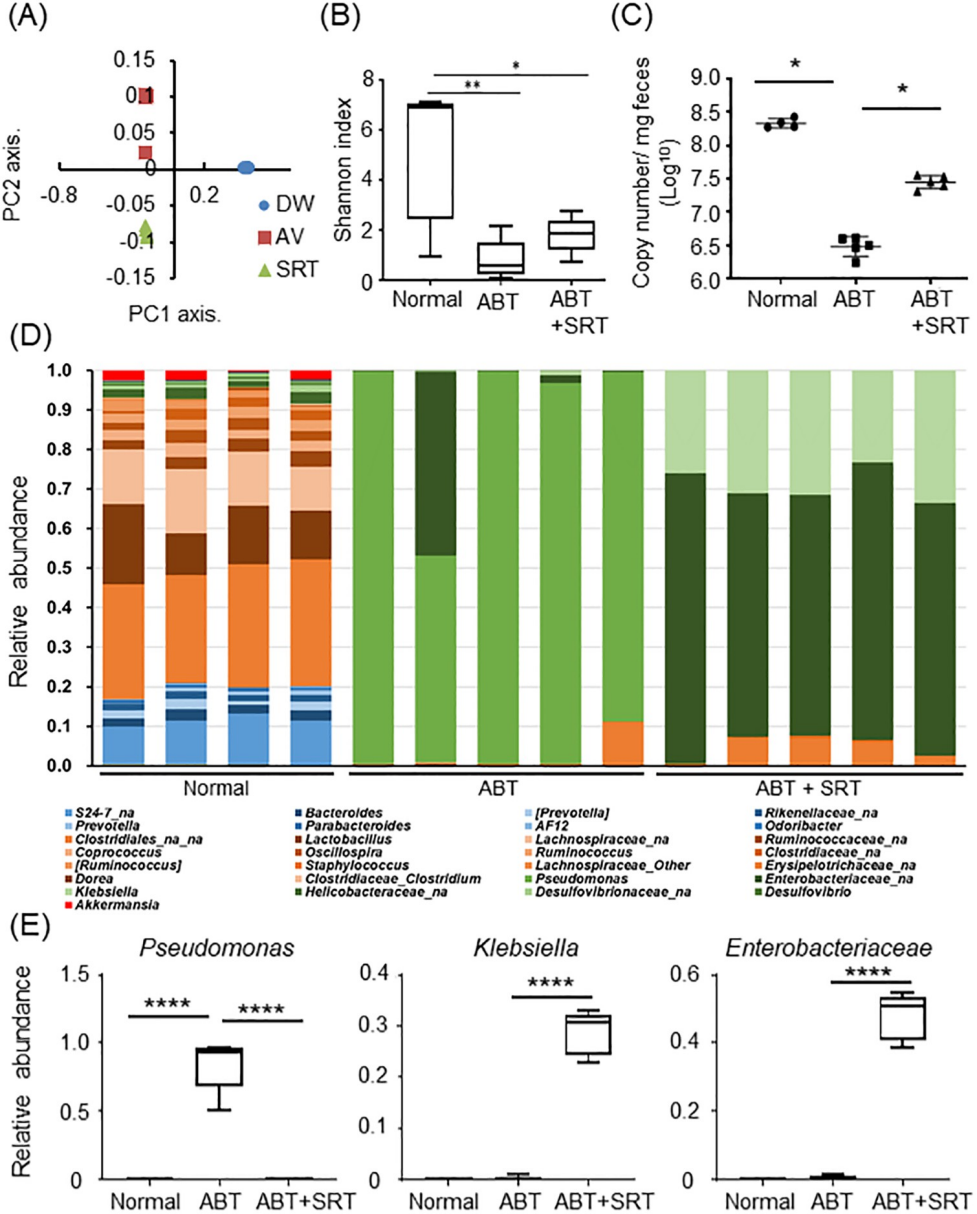
Effect of Saireito on gut microbiota of mice. (A) Visualization of principal coordinates analysis (PCoA) of unweighted Unifrac distances to show differences in bacterial composition. Each point represents the bacterial fecal microbiota in a single sample. (B) α-diversity was measured by the Shannon diversity index of microbiota in fecal samples from different groups (n = 4, normal group. n = 5, ABT and ABT + SRT group). Mann-Whitney *U* test was used to determine statistical significance, * *P* < 0.05, **P < 0.01. (C) DNA in cecum sample was extracted and copy number of rRNA gene was quantified by qPCR. Mann-Whitney *U* test was used to determine statistical significance, * *P* < 0.05. (D) Fecal samples were subjected to 16S ribosomal RNA sequencing to evaluate the composition of gut microbiota. The relative bacterial abundance is shown at the genus level. Each bar shows relative bacterial abundance in individual mice. (E) The relative bacterial abundance is shown at the species level. Data are shown as mean ± SEM (n = 4, normal group. n = 5, ABT and ABT + SRT group) **** *P* < 0.0001.

To evaluate the effects of Saireito treatment on gut microbiota in mice, we performed 16S rRNA sequencing of fecal samples. 16S rRNA genes were sequenced and principal coordinate analysis (PCoA) plots were generated by QIIME 1.9.0 from MiSeq data. PCoA plots showed that each group formed a cluster of different intestinal flora in unweighted UniFrac distances (Fig 6A). As expected, fecal samples from the ABT and ABT + SRT groups had significantly reduced microbial diversity in the rarefaction analysis of alpha diversity (Fig 6B). Next, we quantified the copy number of rRNA genes in fecal samples using qPCR. The copy number of rRNA in fecal samples was significantly decreased in the ABT group compared with the normal group, which was restored by Saireito treatment (Fig 6C). Although Saireito treatment alone did not change the composition of intestinal microbiota at the genus level (S2 Fig), significant changes were seen in the ABT as well as ABT + SRT groups (Fig 6D). *Pseudomonas* were highly abundant in the fecal samples of the ABT group, but not in the normal or ABT + SRT groups (Fig 6D and 6E). *Enterobacteriaceae* and *Klebsiella* were predominant in the ABT + SRT group (Fig 6D and 6E). Interestingly, *Clostridiales* that was absent in the feces of the ABT group reappeared in the ABT + SRT group (Fig 6D). This data indicates that Saireito substantially alters the composition of intestinal microbiota disturbed by antibiotics.

We have previously reported that antibiotic treatment resulted in the perturbation of intestinal metabolites due to intestinal dysbiosis [12]. Thus, we examined the intestinal metabolites in mice treated with Saireito using GC/MS. Administration of antibiotics markedly reduced short-chain fatty acids (SCFAs) such as butyric acid, acetic acid, and propionic acid, which are important for maintaining intestinal homeostasis. On the other hand, formic acid and caproic acid were significantly increased in the feces of the antibiotic-treated mice. In the ABT + SRT group, acetic acid, propionic acid, and valeric acid were slightly, but not significantly, increased, and formic acid and caproic acid were reduced compared to the normal group (S3 Fig). Altered levels of glutamic acid and lactic acid in the ABT group were not affected by Saireito treatment (S3 Fig).

## Discussion

There are a variety of exogenous microorganisms, toxins, and antigens in the intestinal tract. Intestinal epithelial barrier dysfunction might contribute to increased intestinal permeability that allows these substances to penetrate the tissue, enter the bloodstream, and evoke immune responses. This phenomenon has recently been recognized as "leaky gut syndrome" and triggers not only enteritis but also various types of autoimmune diseases [8–11]. In patients with leaky gut syndrome, the physical epithelial barrier is damaged and permeability is increased [9]. The intestinal epithelial barrier consists of intestinal epithelial cells, mucus, tight junctions, and antimicrobial peptides, which contribute to maintaining the functional integrity of the barrier by preventing the permeation of harmful intestinal luminal contents into the body [26]. Furthermore, the balance between cell proliferation and death of intestinal epithelial cells is a critical factor in the maintenance of gut homeostasis [22], and increased apoptosis, in particular, can be a risk factor leading to intestinal epithelial barrier dysfunction [27, 28].

It has been reported that oral administration of antibiotics results in intestinal dysbiosis and leaky gut in humans [4–6]. Mice treated with a single agent of AMPC or cefixime also displayed enhanced intestinal permeability in the colon [29, 30]. Consistent with these reports, we observed dysbiosis of gut microbiota and increased intestinal permeability in the colon of mice treated with ampicillin and vancomycin. Thus, antibiotic-treated mice (A + V-treated mice) appeared to reproduce the pathology of leaky gut caused by dysbiosis.

Saireito, a traditional Japanese herbal medicine, has been shown to exert an anti-inflammatory effect in experimental models of colitis and intestinal mucositis [16, 17]. For example, Kato *et al*. found that Saireito suppressed 5-FU-induced intestinal mucositis in mice by reducing cell apoptosis through inhibition of TNF-α and IL-1β expression [16]. Moreover, Watanabe *et al*. reported that Saireito ameliorated oxazolone-induced colitis, an animal model of IBD, by down-regulating type 2 immune responses [17]. We also found that pretreatment of mice with Saireito suppressed epithelial cell apoptosis and intestinal hyperpermeability induced by antibiotics. When we examined the expression of E-cadherin as well as tight junction proteins (TJPs) such as ZO-1, occludin, and claudin-4, which are known as key components of the physical integrity regulating intestinal permeability [31], we found their levels to be significantly reduced by antibiotic treatment but found they were restored with administration of Saireito. Moreover, the expression of mRNA of TJPs in intestinal epithelial cells was enhanced by Saireito *in vitro*. These results indicate that Saireito protects the physical barrier by inhibiting intestinal epithelial apoptosis and inducing TJPs in intestinal epithelial cells, which prevents leaky gut induced by antibiotic treatment.

In the present study, antibiotic treatment markedly altered the composition of gut microbiota in mice. Consistent with previous reports [29, 30], we observed a decrease in bacterial α-diversity and modifications of β-diversity in the feces of A+V-treated mice, which was significantly improved by Saireito treatment and suggests Saireito exhibits a suppressive effect on dysbiosis in mice. At the genus level, the gut microbiota community in the ABT group was mostly occupied by *Pseudomonas spp*., known as a pathogenic bacterium causing sepsis and antimicrobial-associated diarrhea in humans [32, 33]. *Pseudomonas spp*. also increases intestinal permeability through the induction of IL-1β and TNF-α and exacerbates chronic colitis in IL-10 deficient mice [34, 35]. Furthermore, quorum sensing autoinducer of *Pseudomonas* ablates the extracellular matrix and tight junctions by triggering oxidative stress and apoptosis, and finally disrupts the intestinal epithelial barrier [36]. In this study, we found that *Pseudomonas spp*. was diminished and replaced by the genus *Klebsiella* and *Enterobacteriaceae* in the ABT + SRT group. Given the pathogenesis of *Pseudomonas spp*., the colitis with positive fecal occult bleeding and leaky gut induced by ABPC and VCM might be associated with the dysbiosis accompanied by increased *Pseudomonas spp*. in the colon. In addition, a small population of *Clostridium spp*. that had disappeared with antibiotic treatment reappeared in the ABT + SRT group. Several bacteria belonging to this genus digest dietary fiber and produce SCFAs that are known to enhance the physical integrity by modulating TJPs [37, 38] as well as inhibiting the apoptosis of intestinal epithelial cells [39, 40]. Thus, a reduction of *Clostridium spp*. and metabolic products of SCFAs was implicated in the leaky gut associated with reduced expression of TJPs and enhanced epithelial apoptosis in the colon. Importantly, Saireito supports the growth of beneficial bacteria such as *Clostridium spp*. and reduces potentially harmful bacteria such as *Pseudomonas spp*. in the intestine.

**Fig 7:**
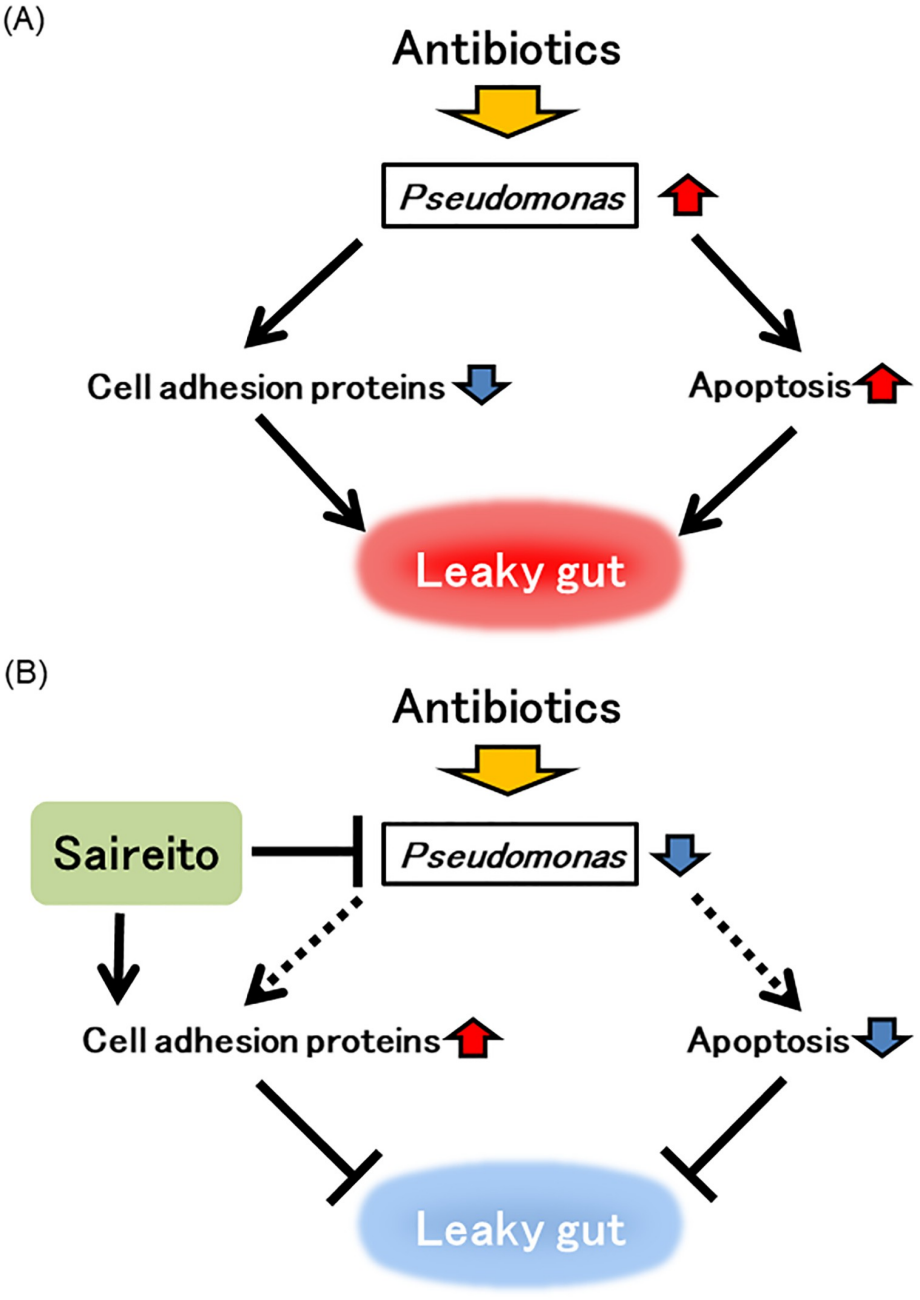
A model for the effect of Saireito on leaky gut associated with antibiotic-induced dysbiosis. The figure summarizes the results of this study. (A) Antibiotic therapy disturbs the normal gut microbiota. *Pseudomonas spp*., which is mostly occupied in the colon of mice treated with antibiotic, disrupts the intestinal epithelial barrier by decreasing the expression of TJPs and increasing epithelial cell apoptosis. (B) Saireito may suppress the growth of *Pseudomonas spp*., enhance the expression of TJPs and inhibit intestinal epithelial apoptosis, which contributes to alleviates leaky gut in antibiotic-treated mice.

In conclusion, Saireito prevented antibiotic-induced dysbiosis, inhibited intestinal epithelial apoptosis and enhanced the expression of TJPs in mice, which contributed to maintaining the physical integrity of the intestinal epithelium and alleviated colitis associated with leaky gut caused by antibiotic-induced dysbiosis (Fig 7). Our findings provide a potentially new therapeutic strategy for gastrointestinal disorders caused by leaky gut.

## Supporting information

S1 Fig Effect of Saireito on the expression of cell adhesion proteins and antimicrobial peptides. (A, B) mRNA analysis of cell adhesion proteins, antimicrobial peptides, and MUC2. Statistical analysis was carried out using one-way ANOVA followed by Tukey’s multiple comparisons test. Data are shown as mean ± SEM (n = 4, each group). * *P* < 0.05, **P < 0.01, ****P < 0.0001, NS: not significant.
(PDF)

S2 Fig Effect of Saireito on gut microbiota of antibiotic-untreated mice. Fecal samples from antibiotic-untreated mice were subjected to 16S ribosomal RNA sequencing to evaluate the composition of gut microbiota. The relative bacterial abundance is shown at the genus level. Each bar shows relative bacterial abundance in individual mice (n = 3, each group).
(PDF)

S3 Fig Effect of Saireito on fecal metabolites in the cecum of mice. (A) The relative concentration of SCFAs in murine feces. (B) The relative concentration of glutamic acid and lactic acid in murine feces. Statistical analysis was carried out using one-way ANOVA followed by Tukey’s multiple comparisons test. Data are expressed as mean ± SEM (n = 4, normal group. n = 5, ABT and ABT + SRT group). * *P* < 0.05, **P < 0.01.
(PDF)

S1 Table Primer sequences used in this study. (PDF)
