# Supplementary figures and images for: Saireito, a Japanese herbal medicine, alleviates leaky gut associated with antibiotic-induced dysbiosis in mice
Source: PLoS One. 2022 Jun 15;17(6):e0269698. doi: 10.1371/journal.pone.0269698 (PMC9200308; doi:10.1371/journal.pone.0269698)

(A)

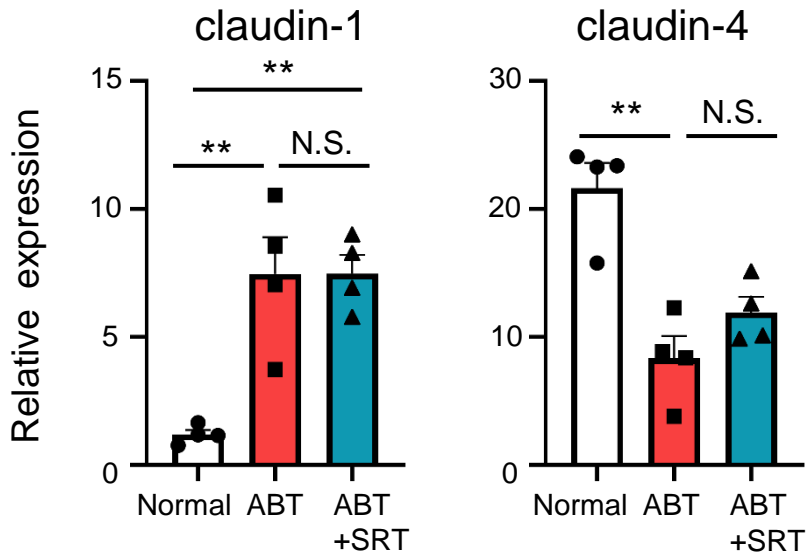

(B)

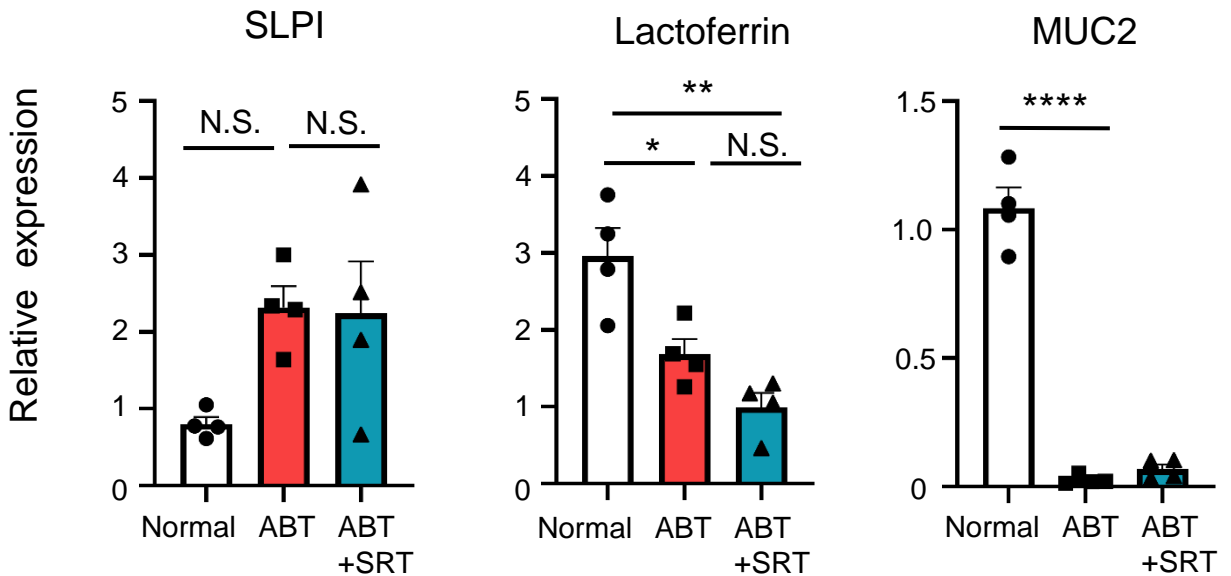

Supplement: S1 Fig — (A, B) mRNA analysis of cell adhesion proteins, antimicrobial peptides, and MUC2. Statistical analysis was carried out using one-way ANOVA followed by Tukey’s multiple comparisons test. Data are shown as mean ± SEM (n = 4, each group). * P < 0.05, **P < 0.01, ****P < 0.0001, NS: not significant. (PDF) [file pone.0269698.s001.pdf]

(A)

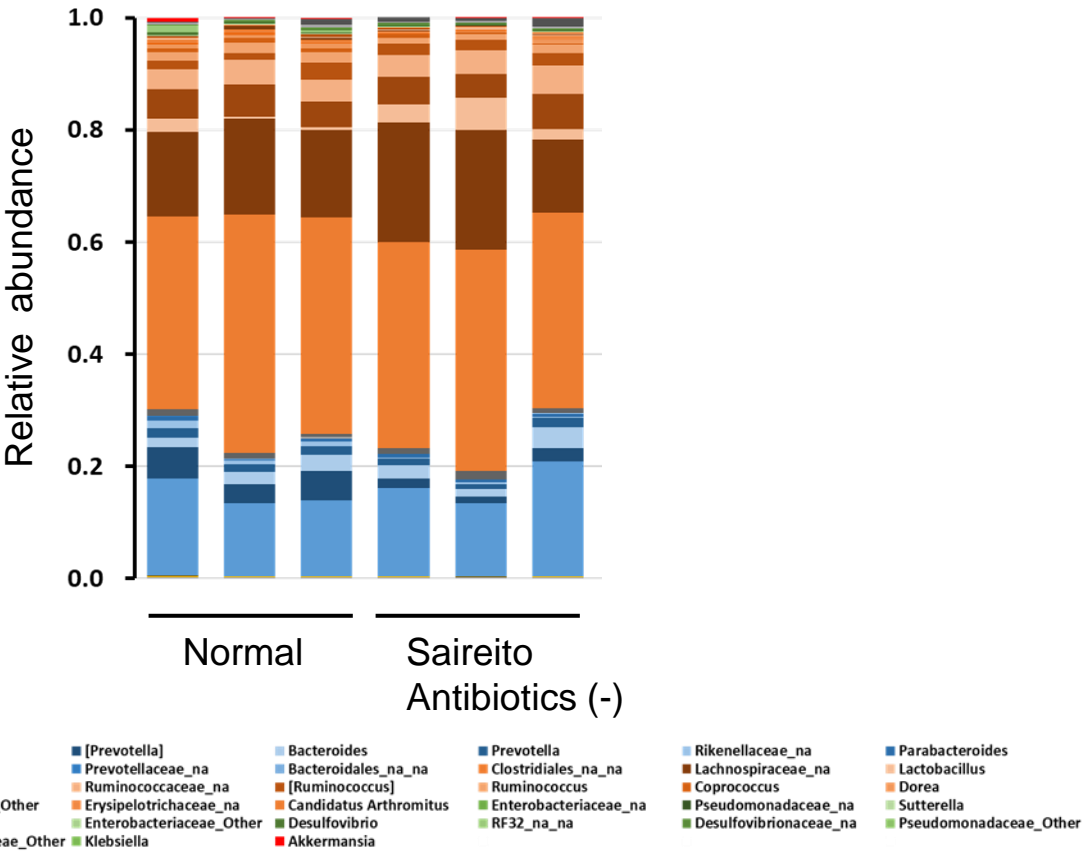

Supplement: S2 Fig — Fecal samples from antibiotic-untreated mice were subjected to 16S ribosomal RNA sequencing to evaluate the composition of gut microbiota. The relative bacterial abundance is shown at the genus level. Each bar shows relative bacterial abundance in individual mice (n = 3, each group). (PDF) [file pone.0269698.s002.pdf]

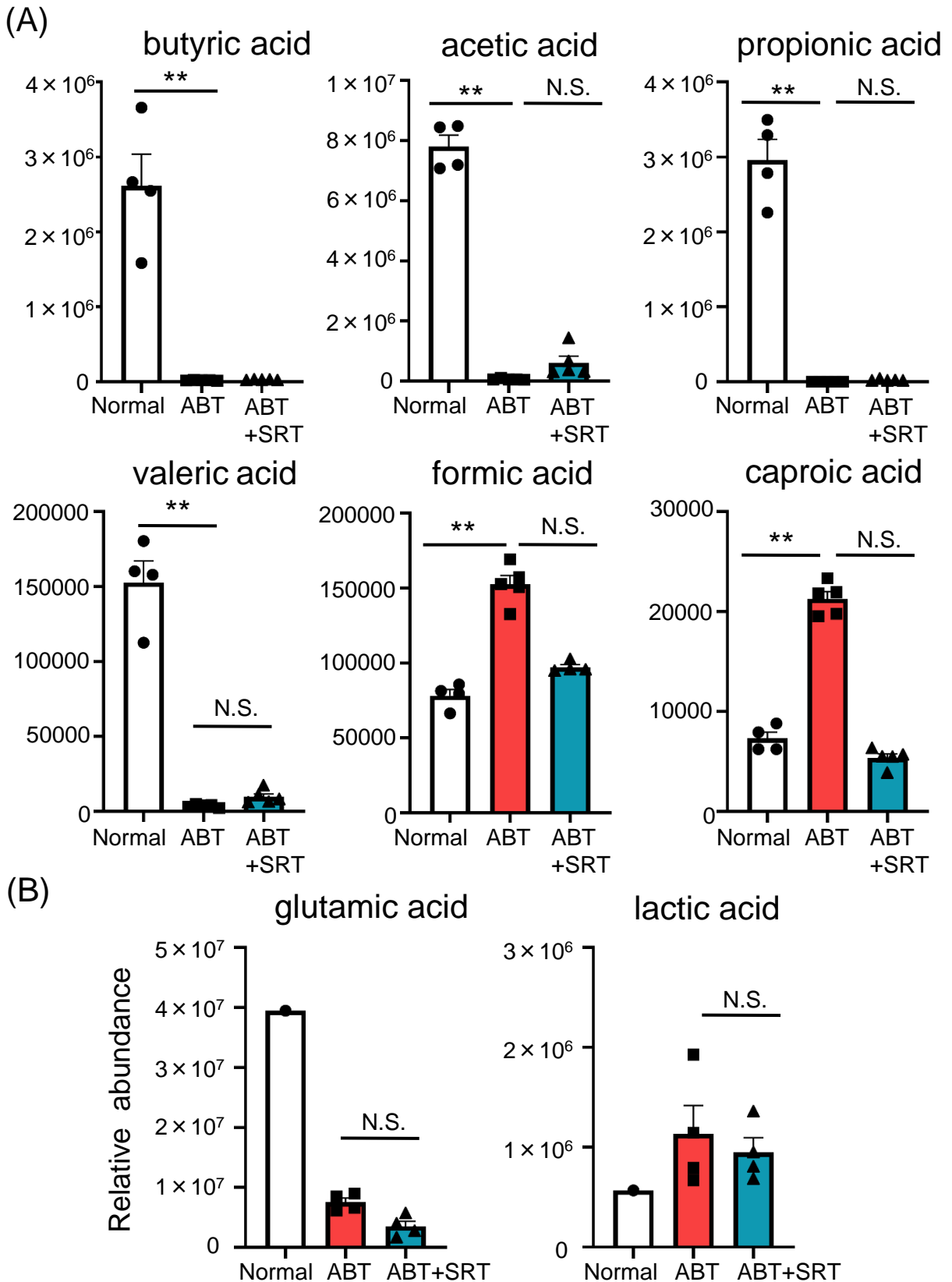

Supplement: S3 Fig — (A) The relative concentration of SCFAs in murine feces. (B) The relative concentration of glutamic acid and lactic acid in murine feces. Statistical analysis was carried out using one-way ANOVA followed by Tukey’s multiple comparisons test. Data are expressed as mean ± SEM (n = 4, normal group. n = 5, ABT and ABT + SRT group). * P < 0.05, **P < 0.01. (PDF) [file pone.0269698.s003.pdf]
