# Supplementary material for: Saireito, a Japanese herbal medicine, alleviates leaky gut associated with antibiotic-induced dysbiosis in mice
Source: PLoS One. 2022 Jun 15;17(6):e0269698. doi: 10.1371/journal.pone.0269698 (PMC9200308; doi:10.1371/journal.pone.0269698)
Supplement: S1 Table — (PDF) [file pone.0269698.s004.pdf]

Target	Forward 5’-3’	Reverse 5’-3’
m β-actin	CTTCCTCCCTGGAGAAGAGCTATGAGC	GCCTAGAAGCACTTGCGGTGCACG
m SLPI	GGCCTTTTACCTTTCACGGTG	TACGGCATTGTGGCTTCTCAA
m lactoferin	TCAAGAAATCCTCCACCCGC	ACACGAGCTACACAGGTTGG
m IL-1β	GAGTGTGGATCCCAAGCAAT	TACCAGTTGGGGAACTCTGC
m IL-6	CCGGAGAGGAGACTTCACAG	CAGAATTGCCATTGCACAAC
m IL-12p40	CCTGAAGTGTGAAGCACCAA	TCAGGGGAACTGCTACTGCT
m IL-13	CCTGGCTCTTGCTTGCCTT	GGTCTTGTGTGATGTTGCTCA
m MUC2	ACATCACCTGTCCCGACTTC	GAGCAAGGGACTCTGGTCTG
m ZO-1	AGCGCCAGGAAGCTATATGA	GCTGAATCGCTTCTTTCAGG
m claudin1	GCTGGGTTTCATCCTGGCTTCT	CCTGAGCGGTCACGATGTTGTC
m claudin4	GGGGATCATCCTGAGTTGTG	CACTGCATCTGACCTGTGCT
m occludin	GCGGAAAGAGTTGACAGTCC	GGCACCAGAGGTGTTGACTT
m E-cadherin	ATCCTCGCCCTGCTGATT	ACCACCGTTCTCCTCCGTA
